# Metachronous Peritoneal Metastases After Adjuvant Chemotherapy are Associated with Poor Outcome After Cytoreduction and HIPEC

**DOI:** 10.1245/s10434-018-6539-x

**Published:** 2018-05-31

**Authors:** Nina R. Sluiter, Koen P. Rovers, Youssra Salhi, Stijn L. Vlek, Veerle M. H. Coupé, Henk M. W. Verheul, Geert Kazemier, Ignace H. J. T. de Hingh, Jurriaan B. Tuynman

**Affiliations:** 1 Department of Surgery, VU University Medical Center, Amsterdam, The Netherlands; 2 Department of Surgery, Catharina Hospital Eindhoven, Eindhoven, The Netherlands; 3 Department of Epidemiology and Biostatistics, VU University Medical Center, Amsterdam, The Netherlands; 4 Department of Medical Oncology, VU University Medical Center, Amsterdam, The Netherlands

## Abstract

**Introduction:**

Cytoreduction and hyperthermic intraperitoneal chemotherapy (HIPEC) improve the survival of colorectal cancer (CRC) patients with peritoneal metastases. Patient selection is key since this treatment is associated with high morbidity. Patients with peritoneal recurrence within 1 year after previous adjuvant chemotherapy are thought to benefit less from HIPEC treatment; however, no published data are available to assist in clinical decision making. This study assessed whether peritoneal recurrence within 1 year after adjuvant chemotherapy was associated with survival after HIPEC treatment.

**Methods:**

Peritoneal recurrence within 1 year after adjuvant chemotherapy, as well as other potentially prognostic clinical and pathological variables, were tested in univariate and multivariate analysis for correlation with primary outcomes, i.e. overall survival (OS) and disease-free survival (DFS). Two prospectively collected databases from the VU University Medical Center Amsterdam and Catherina Hospital Eindhoven containing 345 CRC patients treated with the intent of HIPEC were utilized.

**Results:**

High Peritoneal Cancer Index (PCI) scores were associated with worse DFS [hazard ratio (HR) 1.04, 95% confidence interval (CI) 1.00–1.08, *p* = 0.040] and OS (HR 1.11, 95% CI 1.07–1.15, *p* < 0.001) in multivariate analysis. Furthermore, patients with peritoneal recurrence within 1 year following adjuvant chemotherapy had worse DFS (HR 2.13, 95% CI 1.26–3.61, *p* = 0.005) and OS (HR 2.76, 95% CI 1.45–5.27, *p* = 0.002) than patients who did not receive adjuvant chemotherapy or patients with peritoneal recurrence after 1 year.

**Conclusion:**

Peritoneal recurrence within 1 year after previous adjuvant chemotherapy, as well as high PCI scores, are associated with poor survival after cytoreduction and HIPEC. These factors should be considered in order to avoid high-morbidity treatment in patients who might not benefit from such treatment.

**Electronic supplementary material:**

The online version of this article (10.1245/s10434-018-6539-x) contains supplementary material, which is available to authorized users.

Cytoreductive surgery (CRS) combined with hyperthermic intraperitoneal chemotherapy (HIPEC) is currently the only potentially curative treatment for colorectal cancer (CRC) patients with limited peritoneal metastases (PM).^1^,^2^ This approach increases median survival rates from 12 to 16 months after treatment with systemic chemotherapy alone 3–5 to 33–45 months, translating to a 5-year survival rate of 35%.^4^,^6^,^7^ However, the combination of extensive surgery and HIPEC is associated with relatively high morbidity and mortality rates of 16–64% and 1–5%,^8^–^10^ respectively. Hence, it is of utmost importance to carefully select patients who will benefit most from this treatment.

In recent years, there has been wide interest in the identification of prognostic factors in patients with PM, both clinically 11 and biologically.^12^,^13^ Nevertheless, the lack of randomized controlled trials and the large heterogeneity of published studies limit the use of these variables in clinical practice. The current body of prognostic characteristics include the Peritoneal Cancer Index (PCI; a score for peritoneal tumor burden) and biological tumor characteristics such as primary tumor differentiation.^11^ Some have combined variables to predict outcome after CRS and HIPEC using nomograms such as the Peritoneal Surface Disease Severity Score (PSDSS)^11^ or the Colorectal Peritoneal Metastases Prognostic Surgical Score (COMPASS).^14^,^15^ These characteristics rely heavily on intra- or postoperative findings, whereas selection of HIPEC patients should ideally take place before the start of treatment. Therefore, identification and validation of new prognostic variables that can be assessed preoperatively is warranted.

In clinical practice, patients developing PM despite treatment with adjuvant chemotherapy after primary tumor surgery seem to benefit less from CRS and HIPEC, especially if PM are diagnosed within 1 year after primary tumor resection, or even during chemotherapeutic treatment. Accordingly, several studies exclude patients with PM development or progression despite systemic chemotherapy from CRS and HIPEC.^6^,^16^,^17^ Moreover, certain guidelines encourage consideration of this factor in the CRS and HIPEC selection process; 18,19 however, no data are currently available to support this decision in clinical practice. This study aimed to assess whether peritoneal recurrence within 1 year after adjuvant chemotherapy is associated with poor survival in CRC patients after CRS and HIPEC.

## Methods

### Operative Treatment

Patients from the VU University Medical Center and the Catharina Hospital Eindhoven, two tertiary referral centers, were included in this study. Both hospitals performed CRS and HIPEC according to the same standardized protocol.^7^ Cytoreductive surgery consisted of complete debulking, stripping of the affected peritoneum, and removal of the omentum.^20^ When deemed necessary, multiorgan resections were carried out. Subsequently, if a macroscopic complete resection was achieved, either oxaliplatin (460 mg/m^2^ body surface) or mitomycin C (35 mg/m^2^ body surface) was installed in the peritoneal cavity, with a target temperature of 39–41 °C for 30 or 90 min, respectively.

### Patient Selection and Data Collection

The present study was approved by the Medical Ethics Review Committee of the VU University Medical Center (2018.124). Patients with PM of colorectal adenocarcinoma who underwent surgery with the intent of CRS and HIPEC were considered for inclusion in this study, whereas patients with synchronous metastases and low-grade appendiceal mucinous neoplasms (LAMN), as well as patients without histologically proven PM, were excluded. The following clinicopathological and follow-up data were collected from the records of both institutions: age, sex, American Society of Anesthesiologists (ASA) score, information on comorbidity and primary tumor characteristics, prior treatment, and prior surgical scores (PSS). A PSS of 0 was recorded when there was no history of abdominal surgery or only a biopsy; a PSS of 1 was recorded when abdominal surgery was performed in one of the abdominal regions; and a PSS of 2 was recorded for surgery in two to five regions, and a PSS of 3 for surgery in more than five regions.

Intraoperatively, the extent of peritoneal disease was quantified using the PCI, a numeric score that combines the lesion size (0–3) with the amount of affected abdominopelvic regions (to a maximum of 13) to a score from 0 to 39.^21^,^22^ After completion of CRS, resection outcome was determined according to the maximal size of residual tumor tissue: an R1 (complete) resection was scored when no macroscopically visible tumor was left behind; an R2a resection was scored when the tumor was smaller than 2.5 mm; and an R2b resection was scored when the residual tumor was larger than 2.5 mm.^23^

Postoperatively, hospital complications were documented and scored according to the Common Terminology Criteria for Adverse Events (CTCAE) v4.0 grading system.^24^ Follow-up data, including recurrences and death, were obtained from both hospitals.

To examine whether patients who developed PM after adjuvant chemotherapy had worse outcomes, patients were divided into four groups based on administration of adjuvant chemotherapy (yes vs. no) and time to diagnosis of PM after primary tumor resection (within 1 year vs. after more than 1 year). These categories will be referred to as follows: (1) PM within 1 year without adjuvant chemotherapy; (2) PM after more than 1 year without adjuvant chemotherapy; (3) PM within 1 year after adjuvant chemotherapy; and (4) PM more than 1 year after adjuvant chemotherapy. A diagnosis of PM was based on regular follow-up after resection of the primary tumor, consisting of carcinoembryonic antigen (CEA) measurements, and ultrasound and computed tomography (CT) scans, according to the Dutch guidelines.^25^

### Statistical Analysis

Associations between clinicopathological variables were tested using the Fisher’s exact test or Chi square test for two categorical/dichotomous variables, or the independent *t* test or one-way analysis of variance (ANOVA) for a continuous, normally distributed variable with a dichotomous or categorical variable, respectively. A significant difference was assumed for a *p* value < 0.05 (two-sided test). If necessary, variables were dichotomized to provide a minimum of ten events per category in the survival analysis. Dichotomization was performed on the basis of mean values for continuous variables. Overall survival (OS) and disease-free survival (DFS) were defined as the time (in months) from the date of CRS and HIPEC to the date of death from any cause or date of recurrence, respectively. Univariate associations between OS or DFS and clinicopathological variables that could be determined preoperatively were tested using the Kaplan–Meier method (log-rank test). Variables with a *p* value ≤ 0.1 in univariate survival analysis were included in a multivariate Cox regression analysis. Variable selection in the Cox model was performed using backward selection, with a threshold *p* value of 0.1 for exclusion from the model. Statistical analyses were performed using the Statistical Package for Social Sciences (SPSS) version 23 for Windows (IBM Corporation, Armonk, NY, USA).

## Results

### Baseline Characteristics

In the VU University Medical Center and Catharina Hospital Eindhoven, 345 patients with peritoneally metastasized CRC were treated with the intent of CRS and HIPEC from January 2008 until May 2016. After exclusion of patients with synchronous metastases, other pathology subtypes, and patients without histologically proven PM, 175 patients were selected for further analysis. Table 1 represents the baseline characteristics of all patients.

**Table 1:** Baseline characteristics of all patients

Characteristic	*n* (%)
General characteristics	
All	175
Female sex	93 (53.1)
Age, years	
Mean (SD)	61.7 (10.3)
ASA classification	
I–II	150 (85.7)
III	25 (14.3)
Primary tumor characteristics	
Location	
Colon	159 (90.9)
Rectum	16 (9.1)
Tumor differentiation	
Good/moderate	127 (84.1)
Poor	20 (13.3)
Signet cell	4 (2.6)
Tumor histology	
Adenocarcinoma	138 (81.7)
Mucinous adenocarcinoma	31 (18.3)
*T* stage	
*T*1–3	107 (61.8)
*T*4	66 (38.2)
*N* stage	
*N*0	66 (37.9)
*N*1–2	108 (62.1)
Distant metastases	13 (7.4)
Stage	
1–2	62 (35.8)
3–4	111 (64.2)
Perioperative treatment	
Primary tumor: adjuvant chemotherapy	111 (64.2)
Primary tumor: adjuvant chemotherapy type	
Oxaliplatin	2 (1.8)
Capecitabine	4 (3.6)
CAPOX	71 (64.0)
FOLFOX	14 (12.6)
5-FU	1 (0.9)
Unknown	19 (17.1)
Development of PM	
≤ 1, no chemotherapy	30 (17.1)
> 1 year, no chemotherapy	34 (19.4)
≤ 1 year after chemotherapy	36 (20.6)
> 1 year after chemotherapy	75 (42.9)
HIPEC: neoadjuvant chemotherapy	21 (12.1)
HIPEC: adjuvant chemotherapy	72 (41.4)
Prior surgical score	
0	13 (7.6)
1	8 (4.7)
2	140 (81.9)
3	10 (5.8)
HIPEC characteristics	
Operative procedure	
Open CRS and HIPEC	138 (78.9)
Laparoscopic CRS and HIPEC	3 (1.7)
Open–close	34 (19.4)
PCI	
Mean (SD)	12 (8)
HIPEC chemotherapy	
Mitomycin C	128 (90.8)
Oxaliplatin	13 (9.2)
Resection score	
R1	135 (77.1)
R2a	6 (3.5)
R2b	34 (19.4)
SAE	
Total	94 (53.7)
Grade I: mild	12 (6.9)
Grade II: moderate	35 (20.0)
Grade III: severe	32 (18.3)
Grade IV: life-threatening	13 (7.4)
Grade V: death	2 (1.1)
Reoperation	32 (18.3)

*ASA* American Society of Anesthesiologists, *CAPOX* capecitabine + oxaliplatin, *CRS* cytoreductive surgery, *FOLFOX* leucovorin + 5-FU + oxaliplatin, *HIPEC* hyperthermic intraperitoneal therapy, *PCI* Peritoneal Cancer Index, *PM* peritoneal metastases, *SAE* serious adverse event, *SD* standard deviation, *5*-*FU* 5-fluorouracil

The majority of baseline characteristics did not differ significantly between the group of patients with PM within 1 year after adjuvant chemotherapy and the groups of patients who did not receive adjuvant chemotherapy or were diagnosed with PM after more than 1 year (electronic supplementary Table 1). As expected, lymph node involvement at the time of primary tumor resection was more frequently diagnosed in patients who received adjuvant chemotherapy (*p* < 0.001).

### Oncologic Outcomes

**Fig. 1:**
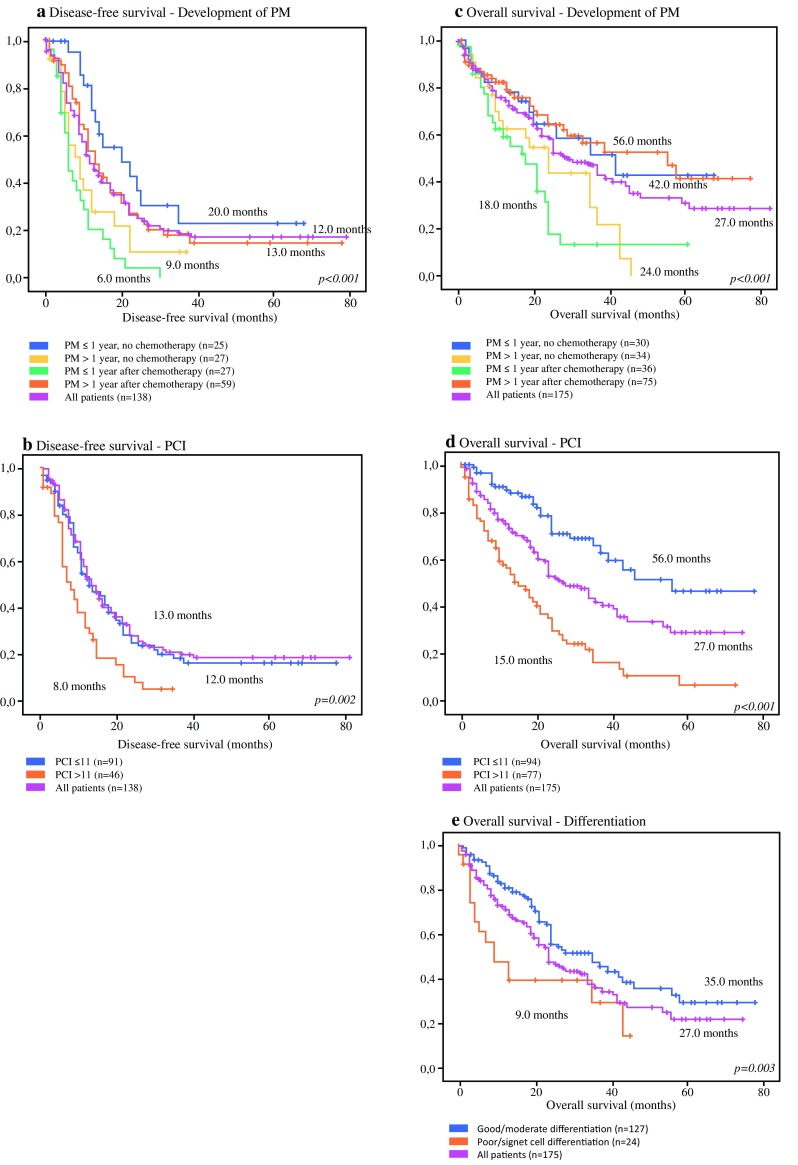
Kaplan–Meier curves of all patients. Graphs (**a**) and (**b**) depict the disease-free survival curves: (**a**) patients with PM within 1 year without chemotherapy, versus PM after more than 1 year without chemotherapy, versus PM within 1 year after chemotherapy, versus PM more than 1 year after chemotherapy; (**b**) patients with a PCI ≤ 11 versus patients with a PCI > 11. Graphs **c–e** depict the overall survival curves: (**c**) patients with PM within 1 year without chemotherapy, versus PM after more than 1 year without chemotherapy, versus PM within 1 year after chemotherapy, versus PM more than 1 year after chemotherapy; (**d**) patients with a PCI ≤ 11 versus patients with a PCI > 11; (**e**) patients with poor/signet cell differentiation versus patients with good/moderate differentiation. *PM* peritoneal metastases, *PCI* Peritoneal Cancer Index

A complete overview of univariate survival analysis is presented in Table 2. Figure 1 depicts the survival curves of the statistically significant variables in univariate analysis.

**Table 2:** Overview of univariate survival analysis

Characteristic	DFS		*p* value^a^	OS		*p* value^a^
	*n*	Median DFS (95% CI)		*n*	Median OS (95% CI)	
General characteristics						
All patients	138	12.0 (8.8–13.2)		175	27.0 (20.6–33.4)	
Sex						
Male	58	11.0 (9.4–12.6)	0.367	82	26.0 (9.7–42.3)	0.768
Female	80	12.0 (8.1–15.9)		93	28.0 (21.6–34.3)	
Age, years						
≤ 60	56	12.0 (8.2–15.8)	0.657	69	28.0 (18.5–37.5)	0.487
> 60	82	11.0 (8.7–13.3)		106	27.0 (17.9–36.1)	
ASA classification						
I–II	121	12.0 (9.7–14.3)	0.309	150	27.0 (20.1–33.8)	0.171
III	17	9.0 (3.8–14.2)		25	16.0 (8.8–23.2)	
Primary tumor characteristics						
Location						
Colon	126	12.0 (10.0–14.0)	0.072	159	28.0 (21.7–34.3)	0.237
Rectum	12	6.0 (4.7–7.3)		16	19.0 (2.9–35.1)	
Differentiation						
Good/moderate	110	12.0 (9.8–14.2)	0.918	127	35.0 (21.6–48.4)	0.003
Poor/signet cell	16	9.0 (7.2–10.8)		24	9.0 (2.0–16.0)	
Histology						
Adenocarcinoma	117	12.0 (9.8–14.2)	0.301	138	29.0 (19.3–38.7)	0.392
Mucinous	19	9.0 (6.2–11.8)		31	23.0 (16.0–30.1)	
*T* stage						
*T*1–3	85	11.0 (8.8–13.2)	0.387	107	24.0 (15.0–33.0)	0.918
*T*4	51	14.0 (9.7–18.3)		66	29.0 (16.7–41.3)	
*N* stage						
*N*0	53	14.0 (6.7–21.3)	0.181	66	35.0 (20.6–49.4)	0.790
*N*1–2	84	11.0 (8.9–13.1)		108	24.0 (19.5–28.5)	
Distant metastases						
No	128	11.0 (8.7–13.3)	0.896	162	28.0 (21.0–35.0)	0.610
Yes	10	11.0 (2.0–20.0)		13	24.0 (18.4–29.6)	
Stage						
Stage 1–2	49	14.0 (6.1–21.9)	0.169	62	35.0 (18.4–51.6)	0.982
Stage 3–4	88	11.0 (9.0–13.0)		112	24.0 (19.8–28.2)	
Perioperative treatment						
Primary tumor: adjuvant chemotherapy						
No	52	12.0 (8.0–16.0)	0.194	64	35.0 (22.8–47.2)	0.658
Yes	86	11.0 (9.3–12.7)		111	24.0 (18.4–29.6)	
Development of PM after primary tumor resection						
≤ 1 year, no chemotherapy	25	20.0 (7.1–32.9)	< 0.001	30	42.0 (17.7–66.4)	< 0.001
> 1 year, no chemotherapy	27	9.0 (4.5–13.5)		34	24.0 (15.9–32.1)	
≤ 1 year after chemotherapy	27	6.0 (4.1–7.9)		36	18.0 (11.7–24.3)	
> 1 year after chemotherapy	59	13.0 (10.2–15.8)		75	56.0 (28.9–83.2)	
HIPEC: neoadjuvant chemotherapy						
No	124	12.0 (10.0–14.0)	0.781	154	27.0 (19.9–34.1)	0.565
Yes	14	9.0 (5.6–12.4)		21	24.0 (8.6–39.4)	
HIPEC: adjuvant chemotherapy						
No	77	11.0 (9.0–13.0)	0.496	102	24.0 (12.7–35.3)	0.167
Yes	61	12.0 (9.6–14.4)		72	28.0 (12.4–43.6)	
Prior surgical score						
0–2	129	11.0 (8.8–13.2)	0.577	161	24.0 (16.8–31.2)	0.075
3	9	21.0 (8.2–33.8)		10	Not reached	
HIPEC/PM characteristics						
PCI						
≤ 11	91	13.0 (9.6–16.4)	0.002	94	56.0 (–)	< 0.001
> 11	46	8.0 (4.9–11.1)		77	15.0 (9.4–20.6)	
HIPEC chemotherapy type						
Mitomycin C	126	11.0 (9.0–13.0)	0.649	128	37.0 (26.3–47.7)	0.922
Oxaliplatin	12	22.0 (0–51.5)		13	29.0 (–)	

*ASA* American Society of Anesthesiologists, *CI* confidence interval, *DFS* disease-free survival, *HIPEC* hyperthermic intraperitoneal therapy, *OS* overall survival, *PCI* Peritoneal Cancer Index, *PM* peritoneal metastases

^a^Log-rank test

### Disease-Free Survival

The median DFS of the whole cohort was 12.0 months (95% CI 8.8–13.2). Patients with a diagnosis of PM within 1 year after adjuvant chemotherapy had a median DFS of 6.0 months (95% CI 4.1–7.9), compared with 20.0 months (95% CI 7.1–32.9) in patients with PM within 1 year without adjuvant chemotherapy, 9.0 months (95% CI 4.5–13.5) in patients with PM after more than 1 year without adjuvant chemotherapy, and 13.0 months (95% CI 10.2–15.8) in patients with PM more than 1 year after adjuvant chemotherapy (*p* < 0.001) (Fig. 1a). Furthermore, patients with a PCI > 11 had a DFS of 8.0 months (95% CI 4.9–11.1), compared with 13.0 months (95% CI 9.6–16.4) in patients with lower PCI scores (*p* = 0.002) (Fig. 1b). Other factors that were considered in univariate survival analysis included gender, sex, age, ASA classification, poor or signet cell tumor differentiation, mucinous tumor type, primary tumor location, primary tumor stage, perioperative treatment, and type of chemotherapy, and were therefore not associated with DFS (Table 2).

### Overall Survival

The median OS of the entire cohort was 27.0 months (95% CI 20.6–33.4). Patients with a diagnosis of PM within 1 year after adjuvant chemotherapy had worse median OS (18.0 months, 95% CI 11.7–24.3) than patients with PM within 1 year without adjuvant chemotherapy (42.0 months, 95% CI 17.7–66.4), patients with PM after more than 1 year without adjuvant chemotherapy (24.0 months, 95% CI 15.9–32.1), and patients with PM more than 1 year after adjuvant chemotherapy (56.0 months, 95% CI 28.9–83.2) (*p* < 0.001). PCI scores > 11 were associated with worse median OS (15 months, 95% CI 9.4–20.6) compared with patients with a PCI ≤ 11 (56.0 months, 95% CI –) (*p* < 0.001; Fig. 1d). Third, a poor or signet cell tumor differentiation was associated with a worse median OS of 9.0 months (95% CI 2.0–16.0), compared with 35.0 months (95% CI 21.6–48.4) in patients with well to moderately differentiated primary tumors (*p* = 0.003) (Fig. 1e).

### Multivariate Analysis

Both high PCI (HR 1.04 for each PCI point, 95% CI 1.00–1.08, *p* = 0.040) and peritoneal recurrence within 1 year after adjuvant chemotherapy (HR 2.13, 95% CI 1.26–3.61, *p* = 0.005) remained significant in the multivariate DFS model (reference category: PM more than 1 year after chemotherapy) (Table 3). In the multivariate OS model, only peritoneal recurrence within 1 year after adjuvant chemotherapy (HR 2.76, 95% CI 1.45–5.27, *p* < 0.002) and high PCI scores (HR 1.11 for each PCI point, 95% CI 1.07–1.15, *p* < 0.001) were associated with worse OS (reference category: PM more than 1 year after chemotherapy) (Table 3). Variables that had a *p* value ≤ 0.1 in univariate survival analysis, but were excluded from the final model after multivariate Cox regression analysis, were primary tumor location (*p* = 0.130) for DFS and primary tumor differentiation (*p* = 0.118) and PSS (*p* = 0.376) for OS.

**Table 3:** Final model resulting from multivariate survival analysis

Variable	Hazard rate (95% CI)	*p* value
Disease-free survival		
PCI	1.04 (1.00–1.08)	0.040
Development of PM		
> 1 year after chemotherapy	Reference	0.001
≤ 1 year, no chemotherapy	0.57 (0.31–1.06)	0.075
> 1 year, no chemotherapy	1.20 (0.67–2.19)	0.535
≤ 1 year after chemotherapy	2.13 (1.26–3.61)	0.005
Overall survival		
PCI	1.11 (1.07–1.15)	< 0.001
Development of PM		
> 1 year after chemotherapy	Reference	0.019
≤ 1 year, no chemotherapy	1.34 (0.62–2.92)	0.454
> 1 year, no chemotherapy	1.89 (0.95–3.76)	0.071
≤ 1 year after chemotherapy	2.76 (1.45–5.27)	0.002

Variables with a *p* value ≤ 0.1 in univariate survival analysis were included in a multivariate Cox regression analysis: (1) primary tumor location, development of PM, and PCI for DFS; (2) primary tumor differentiation, development of PM, PSS, and PCI for OS

*CI* confidence interval, *DFS* disease-free survival, *OS* overall survival, *PCI* Peritoneal Cancer Index, *PM* peritoneal metastases, *PSS* prior surgical score

## Discussion

This prospective cohort study shows that peritoneal recurrence within 1 year after previous adjuvant chemotherapy and a high initial PCI index are the most important risk factors for poor DFS and OS in patients with peritoneally metastasized CRC. This effect was independent of primary tumor stage or tumor differentiation. In 175 patients treated with the intent of CRS and HIPEC in two tertiary referral centers, patients with PM diagnosed within 1 year after adjuvant chemotherapy had worse median DFS (HR 2.13, *p* = 0.005) and OS (HR 2.76, *p* < 0.001) than patients not treated with adjuvant chemotherapy or patients who were diagnosed with PM after more than 1 year in both univariate and multivariate analysis. A high PCI was the only other variable associated with DFS and OS in the multivariate models (HR 1.04, *p* = 0.040; and HR 1.11, *p* < 0.001, respectively).

Cytoreduction and HIPEC is currently the preferred treatment option for patients with PM of CRC,^1^, ^2^ but due to the relatively high morbidity rates associated with this procedure, the need for careful patient selection should be emphasized.^8^–^10^ Preoperative decision making is crucial, warranting practical clinical and pathological prognostic factors. The diagnosis of PM relatively shortly after prior chemotherapy can be assessed preoperatively in contrast to well-established prognostic factors that are obtained after or during the operation, such as PCI, resection scores and combined prognostic scores, including the PSDSS 11 and the relatively novel COMPASS.^14^,^15^ Hence, the selection of patients with early peritoneal recurrence after prior chemotherapy might help identify patients who benefit less from HIPEC, in this way preventing unnecessary exposure to an invasive procedure that may even harm the patient. The association between early peritoneal recurrences after adjuvant chemotherapy and poor outcome could be explained by a more aggressive biological tumor behavior, leading to a poor response to chemotherapy and, subsequently, a poor response to HIPEC treatment.

Several other study groups have already excluded patients with rapid PM development or progression under systemic chemotherapy from CRS and HIPEC.^6^,^16^,^17^ In addition, various guidelines recommend the use of this selection criterion in the workup of potential HIPEC candidates.^18^,^19^ In contrast, two relatively small retrospective studies argued that there is no reason for excluding patients with quick peritoneal relapses after prior chemotherapy from HIPEC. A single-center study including 21 cases found a median OS of 28 months in patients who did not respond to adjuvant chemotherapy, which led the authors to conclude that the survival of this group was comparable with survival rates in other HIPEC patients^6^; however, this study did not include a control group. Another retrospective study compared 19 patients with tumor progression despite neoadjuvant chemotherapy with the same number of patients with stable disease under neoadjuvant chemotherapy, resulting in an insignificant survival difference between these groups.^27^ The relatively small groups in both studies might hamper these outcomes and conclusions.

High PCI emerged as a second characteristic that was associated with poor outcome in the present cohort. This variable is an established prognostic factor and is widely used for the identification of patients with curable peritoneal disease, defined as a PCI ≤ 20.^11^ The present results, supported by the available literature, emphasize the importance of performing a diagnostic laparoscopy to determine PCI indices prior to HIPEC surgery, especially since current imaging techniques lack sensitivity for detection of PM.^28^,^29^ No other variables were associated with DFS and OS in the multivariate models. Importantly, the primary tumor stage was not a significant risk factor for poor DFS and OS.

To our knowledge, this is currently the largest study showing that the development of PM following adjuvant therapy is a poor prognostic factor after treatment with CRS and HIPEC. However, some limitations should be taken into account. First, our data are possibly subjected to selection bias that is associated with cohort studies. Second, based on our results, we can conclude that patients with early peritoneal recurrence after adjuvant chemotherapy have decreased survival; however, there is no solid evidence for an alternative treatment. The effect of second-line chemotherapy in this subgroup of patients, with a poor response to prior chemotherapy, has yet to be proven. Palliative treatment regimens are heterogeneous in this patient group, which makes it hard to gather valid retrospective data from a control cohort. Thereby, new options arise for patients not suitable for HIPEC treatment, including Pressurized IntraPeritoneal Aerosol Chemotherapy (PIPAC). This innovative therapy consists of repetitive intraperitoneal administration of aerosolized chemotherapy and has already been shown to be feasible and safe in end-stage PM originating from several primaries.^30^ For CRC patients, PIPAC with oxaliplatin, whether in combination with systemic chemotherapy or not, showed encouraging results.^31^ Future studies in well-defined populations should demonstrate the potential role of PIPAC in patients with irresectable PM of CRC since the current evidence for PIPAC in this population is scarce.

To provide the answers necessary to take our results into the clinical practice, a prospective study should compare HIPEC with systemic chemotherapy in patients with PM within 1 year after chemotherapy. Until then, CRS and HIPEC may be considered a valid option in this group of patients if the peritoneal tumor burden is limited (PCI ≤ 11). Therefore, whether the limited DFS observed in this cohort of patients with early PM development justifies the high morbidity and mortality rates of the CRS and HIPEC procedure should be carefully considered. Next to identification of clinical prognosticators, future research should focus on identifying molecular tissue and blood-borne characteristics to select patients with biologically favorable tumor characteristics responding best to further aggressive therapies such as CRS and HIPEC. Additionally, identification of biomarkers and clinical prognostic factors could potentially help us optimize the HIPEC procedure, aiming for a personalized treatment rather than the current one-size-fits-all approach. This can be illustrated by selection of the most effective chemotherapeutic drug based on the prediction of individual chemotherapeutic responses,^32^ which may eventually result in a tailored and more robust HIPEC treatment, leading to improved oncologic outcomes in subgroups with relatively poor prognosis.

## Conclusions

The present study found support for considering both PCI and early peritoneal recurrence after adjuvant therapy in patient selection prior to CRS and HIPEC. Prospective trials might help us move forward by confirming or rejecting factors associated with outcome in retrospective studies, ultimately providing clear guidelines to identify the right patients for the right treatment.

## Electronic supplementary material

Below is the link to the electronic supplementary material.

Supplementary material 1 (DOCX 17 kb)
