# Supplementary material for: Metachronous Peritoneal Metastases After Adjuvant Chemotherapy are Associated with Poor Outcome After Cytoreduction and HIPEC
Source: Ann Surg Oncol. 2018 May 31;25(8):2347–56. doi: 10.1245/s10434-018-6539-x (PMC6028868; doi:10.1245/s10434-018-6539-x)
Supplement: Supplementary file 1 — Supplementary material 1 (DOCX 17 kb) [file 10434_2018_6539_MOESM1_ESM.docx]

**Electronic Supplementary Table 1** Baseline characteristic of patients with PM within 1 year without chemotherapy, PM after more than 1 year without chemotherapy, and PM within 1 year after chemotherapy versus PM more than 1 year after chemotherapy

| Development of PM |  | ≤1 year, no chemotherapy | >1 year, no chemotherapy | ≤1 year after chemotherapy | >1 year after chemotherapy | *p*-Value |
| --- | --- | --- | --- | --- | --- | --- |
| *General characteristics* |  |  |  |  |  |  |
| All |  | 30 | 34 | 36 | 75 |  |
| Sex | Male | 16 (53.3) | 13 (38.2) | 16 (44.4) | 37 (49.3) | 0.617* |
|  | Female | 14 (46.7) | 21 (61.8) | 20 (55.6) | 38 (50.7) |  |
| Age, years | Mean [SD] | 60.8 [13.0] | 63.7 [11.7] | 61.1 [8.1] | 61.4 [9.6] | 0.655*** |
| ASA classification | ASA I-II | 24 (80.0) | 25 (73.5) | 32 (88.9) | 69 (92.0) | 0.053** |
|  | ASA III | 6 (20.0) | 9 (26.5) | 4 (11.1) | 6 (8.0) |  |
| *Primary tumor characteristics* | | | | | | |
| Location primary tumor | Colon | 25 (83.3) | 27 (79.4) | 34 (94.4) | 73 (97.3) | 0.005** |
|  | Rectum | 5 (16.7) | 7 (20.7) | 2 (5.6) | 2 (2.7) |  |
| Tumor differentiation | Good/moderate | 23 (88.5) | 22 (75.9) | 29 (82.9) | 53 (86.9) | 0.537** |
|  | Poor/signet cell | 3 (11.5) | 7 (24.1) | 6 (17.1) | 8 (13.1) |  |
| Tumor histology | Adenocarcinoma | 25 (83.3) | 27 (81.8) | 27 (79.4) | 59 (81.9) | 0.983* |
|  | Mucinous | 5 (16.7) | 6 (18.2) | 7 (20.6) | 13 (18.1) |  |
| T stage | T1–3 | 18 (60.0) | 26 (78.8) | 19 (52.8) | 44 (59.5) | 0.142* |
|  | T4 | 12 (40.0) | 7 (21.2) | 17 (47.2) | 30 (40.5) |  |
| N stage | N0 | 21 (70.0) | 27 (79.4) | 5 (13.9) | 13 (17.6) | <0.001* |
|  | N1–2 | 9 (30.0) | 7 (20.6) | 31 (86.1) | 61 (82.4) |  |
| Distant metastases | No | 28 (93.3) | 31 (91.2) | 34 (94.4) | 69 (92.0) | 0.517** |
|  | Yes | 2 (6.7) | 3 (8.8) | 2 (5.6) | 6 (8.0) |  |
| *Perioperative treatment* | | | | | | |
| HIPEC: neoadjuvant chemotherapy | No | 26 (86.7) | 28 (82.4) | 32 (88.9) | 68 (90.7) | 0.647** |
|  | Yes | 4 (13.3) | 6 (17.6) | 4 (11.1) | 7 (9.3) |  |
| HIPEC: adjuvant chemotherapy | No | 13 (44.8) | 17 (50.0) | 22 (61.1) | 50 (66.7) | 0.146* |
|  | Yes | 16 (55.2) | 17 (50.0) | 14 (38.9) | 25 (33.3) |  |
| Prior surgical score | 0–2 | 29 (100.0) | 31 (93.9) | 33 (94.3) | 68 (91.9) | 0.531** |
|  | 3 | 0 | 2 (6.1) | 2 (5.7) | 6 (8.1) |  |
| *HIPEC characteristics* |  |  |  |  |  |  |
| Operative procedure | CRS+HIPEC | 25 (83.3) | 28 (82.4) | 27 (75.0) | 61 (81.3) | 0.829* |
|  | Open–close | 5 (16.7) | 6 (17.6) | 9 (25.0) | 14 (18.7) |  |
| PCI | Mean [SD] | 10.8 [6.5] | 14.3 [9.1] | 14.1 [8.8] | 10.7 [8.1] | 0.062*** |
| Resection score | R1 | 21 (70.0) | 28 (82.4) | 26 (72.2) | 60 (80.0) | 0.539* |
|  | R2 | 9 (30.0) | 5 (17.6) | 10 (27.8) | 15 (20.0) |  |
| SAE | No | 18 (60.0) | 15 (44.1) | 14 (38.9) | 35 (46.7) | 0.375* |
|  | Yes | 12 (40.0) | 19 (55.9) | 22 (61.1) | 40 (53.3) |  |
| Grade complications | I–II | 6 (50.0) | 7 (36.8) | 10 (45.5) | 24 (61.5) | 0.331* |
|  | III–IV | 6 (50.0) | 12 (63.2) | 12 (54.5) | 15 (38.5) |  |
| Reoperation | No | 23 (76.7) | 29 (85.3) | 27 (75.0) | 64 (85.3) | 0.469* |
|  | Yes | 7 (23.3) | 5 (14.7) | 9 (25.0) | 11 (14.7) |  |

Data are expressed as *n*(%) unless otherwise specified

*ANOVA* analysis of variance, *ASA* American Society of Anesthesiologists, *CRS* cytoreductive surgery, *HIPEC* hyperthermic intraperitoneal therapy, *PCI* Peritoneal Cancer Index, *SAE* serious adverse event, *SD* standard deviation

* One-way ANOVA

** Exact test

*** Independent *t*-test
